# Hyperbaric oxygen promotes both the proliferation and chemosensitization of glioblastoma cells by inhibiting HIF1α/HIF2α-ABCG2

**DOI:** 10.3389/fnmol.2025.1584407

**Published:** 2025-04-30

**Authors:** Sheng Gong, Pan Wang, Bin Liao, Lu Zhao, Nan Wu

**Affiliations:** Department of Neurosurgery, Chongqing Research Center for Glioma Precision Medicine, Chongqing General Hospital, Chongqing University, Chongqing, China

**Keywords:** hyperbaric oxygen, HIF1α, HIF2α, ABCG2, chemosensitization, stemness

## Abstract

**Introduction:**

Hyperbaric oxygen enhances glioma chemosensitivity, but the mechanism remains unclear. Hypoxia is common in gliomas, and as the main effector molecules of hypoxia, HIF1α and HIF2α promote the malignant progression by inhibiting cell apoptosis and maintaining stemness. ABCG2 is a marker protein of tumor stem cells and drug efflux transporter protein. This study aims to reveal the detailed mechanism of hyperbaric oxygen promote both proliferation and chemosensitization.

**Methods:**

Under hyperbaric oxygen and hypoxic conditions, we investigated the differences in cell cycle, proliferation, apoptosis, LDH release, and the expression of proteins and mRNA. We also conducted studies on transcriptional regulation and performed *in vivo* experiments.

**Results:**

It revealed that under hypoxic conditions, HIF1α, HIF2α, and ABCG2 are highly expressed, and both HIF1α and HIF2α promote ABCG2 expression. After hyperbaric oxygen treatment, the expression of HIF1α, HIF2α, and ABCG2 decreased, both cell proliferation and chemosensitivity increased. After knocking out HIF1α and HIF2α, cell proliferation and chemosensitivity increased, but the expression of stem cell marker proteins decreased. ChIP-qPCR revealed that HIF1α and HIF2α target the ABCG2 promoter. Gain-and loss-of-function experiments suggested that ABCG2 can promote the expression of stem cell marker proteins, inhibit cell apoptosis, and promote tumor progression.

**Conclusion:**

This study confirmed that hyperbaric oxygen can inhibit ABCG2 expression through HIF1α and HIF2α, thereby promoting the proliferation and chemosensitization of gliomas.

## Introduction

Glioma is the most malignant tumor of the brain and has a poor prognosis due to its aggressive growth, unclear tumor boundary and radiochemotherapy resistance (Paolillo et al., 2018; Huang et al., 2018). The survival period of most patients is only 12–24 months (Paolillo et al., 2018; Arienti et al., 2021; Alpuim Costa et al., 2022). Previous studies have shown that a hypoxic microenvironment can inhibit apoptosis and maintain the stemness of glioma stem cells (GSCs), which is an important reason for the malignant progression and poor prognosis of gliomas (Wang et al., 2021a; Wang et al., 2020; Heddleston et al., 2012). Hyperbaric oxygen (HBO) therapy is a treatment method in which pure oxygen is breathed in via a pressure chamber. This treatment method can transport high concentrations (100%) of oxygen to the body at a level higher than normal atmospheric pressure, thereby improving the oxygen status of various parts of the body, promoting healing and reducing inflammation. Therefore, hyperbaric oxygen (HBO) therapy seems to be an ideal way to alleviate the hypoxic microenvironment. Although there are many controversies about the effect of HBO therapy alone on glioma, there is a general consensus that HBO therapy combined with temozolomide (TMZ) treatment can increase chemotherapy sensitivity and effectively inhibit the growth of gliomas (Shi et al., 2023; Yuen et al., 2023). However, there are few reports on the specific mechanism involved. HIF1α and HIF2α, the main effector molecules of hypoxia, play important roles in the hypoxic microenvironment to promote the malignant progression of gliomas (Wang et al., 2020; Kumar et al., 2023; Wang et al., 2022; Lin et al., 2024). The ATP-binding cassette subfamily G member 2 (ABCG2) is a transmembrane protein that serves as a drug efflux transporter. It can reduce intracellular drug accumulation, thus diminishing the effects of drugs and playing a significant role in tumor multidrug resistance (Dean et al., 2022; Dean et al., 2005; Gong et al., 2011). Meanwhile, ABCG2 is a marker protein of GSCs, that contributes to chemotherapy resistance (Kathawala et al., 2020; Xie et al., 2020; Zhang et al., 2018). Our previous studies revealed that the expression levels of HIF1α, HIF2α, and ABCG2 decreased after HBO treatment. It is still unknown whether there is a certain relationship between the above proteins under HBO treatment conditions and whether HBO has an impact on the chemotherapy sensitivity of glioma cells. Therefore, we conducted a series of experiments to figure out whether the HBO treatment affects tumor chemotherapy sensitivity and proliferation by inhibiting HIF1α/HIF2α-ABCG2.

## Materials and methods

### Public data collection

GEPIA^1^ and CGGA^2^ data were used to analyze the gene expression correlation between HIF1α/HIF2α and ABCG2. The IVY Glioblastoma Atlas Project^3^ was used to analyze the mRNA expression of HIF1α, HIF2α, and ABCG2 in the leading edge region (LE) and microvascular proliferation of the cellular tumor region (CTmvp).

### Cell isolation and cell culture

The primary cells used in this study were obtained from a clinical surgical tumor sample with pathological information of WHO IV, IDH1/2 wild type, which was called GBM. The tissues were digested with 0.2% papain (HyClone, United States) and 10 U/mL DNase I (Sigma, United States) for 50–60 min at 37°C. The erythrocytes were lysed with ACK lysis buffer (Beyotime, China) and then filtered through a 100 μm cell filter. GBM cells were cultured in DMEM/F12 medium supplemented with B27 (Invitrogen), EGF (20 ng/mL), and bFGF (20 ng/mL), and the number of passages was limited to no more than 10. The U87 and A172 cell lines were cultured in DMEM/F12 (HyClone, United States) supplemented with 10% FBS (Gibco, United States). The cultures were incubated in 21% O_2_, 5% CO_2_ at 37°C. The cells were free of contamination.

### Cell treatments

Hypoxia in our experiments was considered 1% O_2_ under normal atmospheric pressure (HYP), while 100% O_2_ at 2.5 ATM was considered hyperbaric oxygen (HBO). For mRNA expression detection and hypoxia probe experiments, cells were cultured under HYP conditions for 12 h. In the HBO group, after 12 h of culture under HYP conditions, the cells were further cultured for 2 h in a HBO environment. For immunofluorescence (IF), LDH release, proliferation, and flow cytometry (FCM) assays, both the HYP group and the HBO group were cultured under 1% O_2_ conditions for 3 days and treated with TMZ (400 μM). However, cells in the HBO group were exposed to HBO for 2 h every day.

### Immunofluorescence analysis

HIF1α, HIF2α, ABCG2, CD133, and CD15 were detected in U87 cells following exposure to HYP or HBO by IF. The cells were fixed in 4% paraformaldehyde at 4°C for at least 30 min, washed with PBS 3 times for 5 min each, permeabilized with 0.5% Triton X-100 (Sigma, United States) for 10 min and washed with PBS. The samples were blocked with goat serum (BOSTER, China) for 30 min, incubated overnight with primary antibody and washed with PBS (see Supplementary Table S1 for the antibody). The samples were then incubated with a secondary antibody conjugated to Alexa Fluor 555 (Beyotime, China) for 2 h. The samples were washed, and Antifade Mounting Medium with DAPI (Beyotime, China) were added. An inverted fluorescence microscope was used for imaging.

### Flow cytometry analysis

The cells were digested with 0.25% trypsin to obtain cell precipitates for cell counting. The cell density was adjusted to approximately 1 × 10^6^ cells/mL, and 100 μL of the cell suspension was centrifuged at 1,000 rpm for 5 min. The supernatant was discarded, and the cells were resuspended by adding 100 μL of Annexin V Binding Buffer, 5 μL of Annexin V-FITC and 5 μL of PI solution according to the instructions of the FITC Annexin V Apoptosis Detection Kit I (BD Pharmingen, United States) and incubated at room temperature in the dark for 15 min. Cell apoptosis was detected. Approximately 5 × 10^5^ cells were removed, the supernatant was discarded, and 1 mL of 75% ethanol precooled at 4°C was added to fix the cells for 24 h. The cells were then centrifuged at 1,000 rpm for 5 min, after which the supernatant was discarded. The cells were resuspended in 1 mL of PBS and centrifuged at 1,000 rpm for 5 min, after which the supernatant was discarded. The cells were resuspended in 200 μL of PBS, and then 5 μL of PI and 5 μL of RNase were added according to the instructions of the Cell Cycle Analysis Kit (Beyotime, China). The cells were incubated at 37°C for 30 min and then stored at 4°C. The cell cycle was detected within 24 h. The cells were cultured with 10 μM BrdU for 1 h, digested, fixed with paraformaldehyde at 4°C for 30 min, acidified with 2 M HCL for 30 min, alkali-neutralized with 0.1 M sodium tetraborate for 10 min, and incubated with an anti-BrdU antibody (Abcam, United States) for 30 min. Then, the cells were incubated with an anti-rat IgG (H + L) secondary antibody (CST, United States) 30 min, resuspended in PBS, and incubated with 5 μl of PI. The percentage of BrdU-stained cells was measured.

### Hypoxyprobe^™^-1 Kit

The Hypoxyprobe^™^-1 Kit (Hypoxyprobe, United States) was used to examine the hypoxic conditions of the U87 cells. Hypoxyprobe^™^-1 was added to the culture medium at a concentration of 100 μmol/mL, and the cells were continuously cultured in HYP or HBO for 1 h. Afterward, the cells were fixed with 4% paraformaldehyde for 30 min and then subjected to the immunofluorescence staining. Hypoxia in mouse tumor tissues was detected by administering a Hypoxyprobe^™^-1 solution (60 mg/kg) via tail vein injection. After a one-hour period, brain tissue samples were harvested and processed following the manufacturer’s instructions for fixation, staining, and other necessary procedures. Images were subsequently captured via microscope.

### Western blotting

Cells were lysed using RIPA buffer (with PMSF) (Beyotime, China), and total protein was extracted by centrifugation. After the protein concentration was detected with a BCA Protein Assay Kit (Beyotime, China), SDS-PAGE electrophoresis was carried out, after which the proteins were transferred to membranes at 200 mA for 2 h; the membranes were blocked with 5% skim milk powder for 2 h, incubated with primary antibody overnight (see Supplementary Table S2 for the antibody), washed with TBST, incubated with HRP-labeled secondary antibody (Beyotime, China), incubated at room temperature for 2 h, and washed with TBST. Enhanced chemiluminescence was used for visualization.

### Real-time quantitative polymerase chain reaction

Total RNA was extracted using the RNASimple Total RNA Kit (TIANGEN, China). Reverse transcription was performed with MightyScript First Strand cDNA Synthesis Master Mix (Sangon, China). Amplification was performed using 2X SG Fast qPCR Master Mix (Sangon, China) under the following conditions: predenaturation at 95°C for 3 min; 40 cycles of denaturation at 95°C for 3 s; and annealing/extension/data acquisition at 60°C for 20 s (see Supplementary Table S3 for the primer sequences). The data were collected on a Bio-Rad CFX96 real-time PCR instrument (Bio-Rad, United States).

### CCK-8 assay

CCK-8 assays were used to assess cell proliferation in the absence or presence of temozolomide (TMZ, 400 μM) under hypoxia or HBO treatment conditions. Then, 100 μL of the suspension was mixed according to the ratio of 10 μL of CCK-8 reagent and 90 μL of DMEM/F12 (HyClone, United States) supplemented with 10% fetal bovine serum (FBS, Gibco, United States) and added to the 96-well plates. The culture plates were cultured in HYP or HBO for 2 h, and the absorbance at 450 nm was detected by a Multiscan Spectrum (Thermo Scientific, United States).

### LDH release

The LDH Cytotoxicity Assay Kit (Beyotime, China) was used to examine the release of LDH in culture. The supernatant was centrifuged at 400 × g for 5 min, 120 μL was added to a new 96-well plate, and 60 μL of LDH detection solution was added to each plate, and incubated at room temperature in the dark for 30 min. The absorbance at 490 nm was detected with a Multiscan Spectrum (Thermo Scientific, United States). The formula for calculating cytotoxicity was as follows: cytotoxicity (%) = (treatment group − control group)/(maximum cell enzyme activity − control group) × 100.

### ChIP-qPCR

Chromatin immunoprecipitation was performed using the Pierce Agarose ChIP Kit (Thermo Scientific, United States). According to the instructions, the experimental procedure included crosslinking, cell pellet isolation, lysis and MNase digestion, immunoprecipitation, IP elution, and DNA recovery. The target gene fragments were obtained and used as templates, and primers were designed and synthesized primers based on the prediction of the ABCG2 promoter region (see Supplementary Table S4 for the primer sequences) for qPCR experiments. The data were collected on a CFX96 real-time PCR instrument (Bio-Rad, United States).

### Dual-luciferase reporter assay

To investigate HIF1α/2α-mediated transcriptional regulation of ABCG2, a dual-luciferase reporter (DLR) system was employed. The ABCG2 promoter region (−2,000 to −1) was cloned into the pGL3 vector to generate the wild-type (Wt) construct. Mutations were introduced at the predicted HIF1α and HIF2α binding sites (completed by Shanghai Sangon Biotech, GCGT → AATG), generating the mutant (Mut) construct. U87 cells were seeded in 24-well plates (5 × 10^4^ cells/well), Wt/Mut reporter plasmid were co-transfected with PRL vector (Promega, United States) using Lipofectamine 2000. Cells were cultured in HYP or NOR conditions for 24 h. Luciferase activity was quantified according to the instructions of Dual-Luciferase Reporter Assay Kit (Promega E1910). Relative activity was calculated as Firefly/Renilla luminescence ratio.

### *In vivo* study

BALB/c-nude mice (male, 4–6 weeks old) were used in this study. The groups included normoxia + DMSO, normoxia + TMZ, HBO + DMSO, HBO + TMZ, normoxia-vector + TMZ, normoxia-ABCG2-KO + TMZ, HBO-vector + TMZ, and HBO-ABCG2-OE + TMZ (15 mice in each group). GBM cells, empty cells, ABCG2-KO cells, and ABCG2-OE cells (5 × 10^4^) were implanted into the brains of mice and fed under an environment of 21% O_2_. HBO and TMZ were administered during the period of 3–17 days. The HBO treatment required a slow pressurization and decompression process of 10–20 min to adjust to the change in pressure. Then, the pressure was increased to 2.5 ATM under 100% O_2_ for 90 min, followed by slow decompression. TMZ (2 mg/kg) was injected into the enterocoelia within 30 min after decompression. *In vivo* imaging was performed after 3 weeks, three mice in each group were selected for tumor collection, and the tumor weight was measured. Real-time quantitative polymerase chain reaction (RT-qPCR) and western blotting (WB) were used to detect the differences in the mRNA and protein expression of HIF1α and HIF2α in tumor tissues under different oxygen conditions. The remaining mice were used for survival analysis.

Empty vector cells, HIF1α-KO cells, HIF2α-KO cells, and dual HIF1α/HIF2α-KO cells (5 × 10^4^) were implanted into the brains of BALB/c-nude mice and reared in 21% O_2_. TMZ (2 mg/kg) was administered during the period of 3–17 days. After 3 weeks of feeding, MRI was performed, and survival time was statistically analyzed.

### Statistical analysis

Statistical analysis was performed using GraphPad Prism 8 software. All experiments were performed at least three times. The data are displayed as mean ± standard deviation. All variables were assessed for normality using the Shapiro–Wilk test. Student’s *t*-test was used to analyze the significance of differences between two groups, one-way ANOVA was used to compare multiple groups, the log-rank test was used for survival analysis, and Pearson’s analysis was used to determine the correlation between HIF1α/HIF2α and ABCG2. *p* < 0.05 was considered to indicate statistical significance.

## Results

### Hyperbaric oxygen promotes glioma cell proliferation but increases chemosensitivity

**Figure 1:**
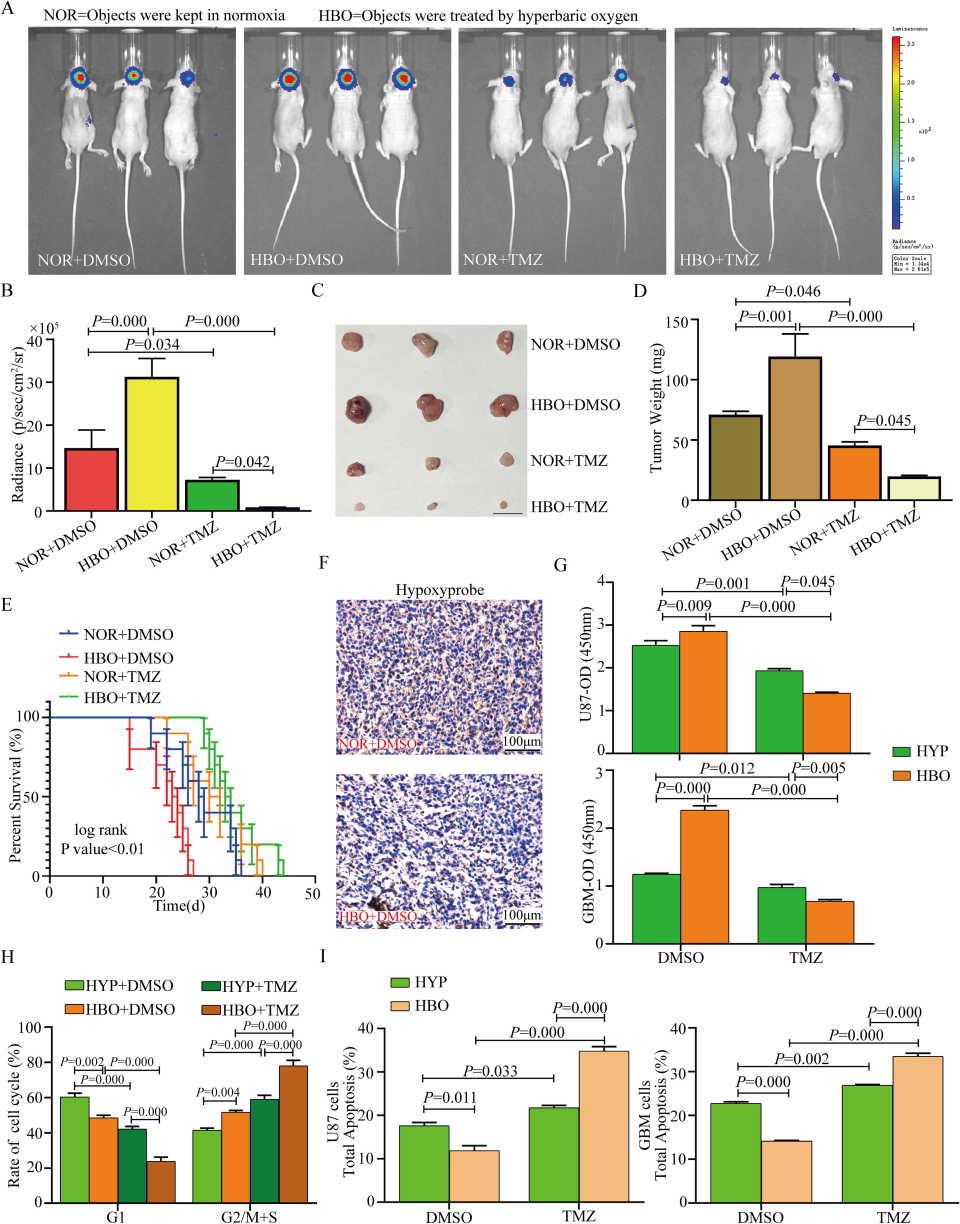
HBO promotes glioma cell proliferation but promotes chemosensitization. **(A–E)** *In vivo* analysis revealed that HBO without TMZ treatment promoted tumor growth, shortened survival time, while combined HBO with TMZ treatment reduced tumor size and weight and prolonged survival time. **(F)** Hypoxyprobe^™^-1 revealed that hypoxia was a prevalent phenomenon in mouse gliomas. **(G)** CCK-8 assays showed that HBO promoted the proliferation of U87 and glioblastoma (GBM) cells compared with that under hypoxic conditions, but proliferation was significantly reduced when HBO was combined with TMZ. **(H)** Cell cycle analysis revealed that compared with hypoxia, HBO induced G2/M + S phase arrest in U87 cells, and TMZ treatment strengthened the cell cycle blockade effect. **(I)** Treatment with HBO alone inhibited the apoptosis of U87 and GBM cells, and the total percentage of apoptosis cells significantly increased after treatment with both HBO and TMZ. HBO, hyperbaric oxygen; NOR, normoxia; HYP, hypoxia; TMZ, temozolomide.

To explore the impact of hyperbaric oxygen treatment on the proliferation and chemotherapy sensitivity of glioma, we undertook an *in vivo* study in nude mice, it revealed that HBO treatment in the absence of TMZ promoted tumor growth but that HBO therapy combined with TMZ treatment significantly reduced tumor size and weight (Figures 1A–D). *In vivo* survival analysis revealed that mice that underwent HBO treatment without TMZ treatment had the shortest survival period, with a median survival time of 23.5 ± 4.37 days, but HBO combined with TMZ treatment significantly prolonged the survival time of mice to the median survival time of 33.5 ± 5.23 days (Figure 1E). Hypoxyprobe^™^-1 exhibited significantly higher expression in tumor tissues from mice maintained under normoxic conditions than in those from mice kept under hyperbaric oxygen conditions, suggesting that hypoxia is a common phenomenon in mouse gliomas (Figure 1F). To explore the impact of hyperbaric oxygen treatment on the proliferation and chemotherapy sensitivity of glioma cells, we conducted *in vitro* studies on glioma cells, the results of the CCK-8 assay showed that HBO promoted the proliferation of U87, GBM and A172 cells compared with that under hypoxic conditions, while after treatment with TMZ, the proliferation of the HBO group was significantly reduced (Figure 1G; Supplementary Figure S1A). The cell cycle analysis results showed that there was a greater proportion of cells in the G2/M + S phase in the HBO-treated group (51.62% ± 1.11, 77.94% ± 3.22) than in the hypoxia-treated group (41.49% ± 1.2, 59.08 ± 2.21) (Figure 1H). Moreover, compared with that in hypoxic cells, apoptosis under HBO treatment conditions was reduced, and apoptosis increased under treatment with both HBO and TMZ (Figure 1I; Supplementary Figure S1B).

### Hyperbaric oxygen inhibits the expression of HIF1α, HIF2α, CD133, and CD15

**Figure 2:**
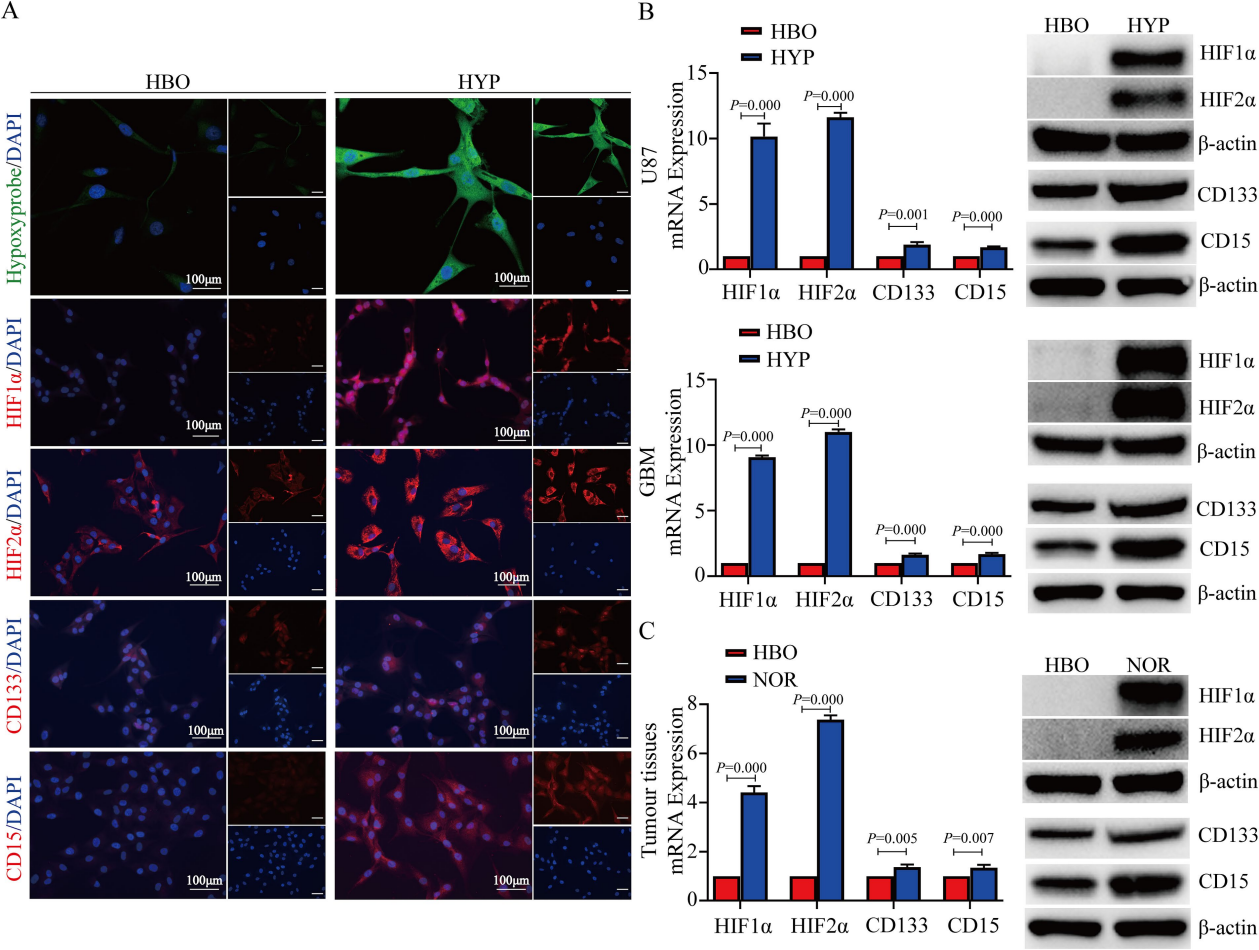
Hyperbaric oxygen inhibits the expression of HIF1α, HIF2α, CD133, and CD15. **(A)** Hypoxyprobe, HIF1α, HIF2α, CD133, and CD15 were more highly expressed in U87 cells under hypoxia than in those under HBO treatment. **(B)** HIF1α, HIF2α, CD133, and CD15 were more highly expressed in U87 and GBM cells under hypoxia than under HBO treatment conditions according to the RT-qPCR and western blotting results. **(C)** The RT-qPCR and western blotting results showing that HIF1α and HIF2α were more highly expressed in tumor tissues from mice reared under normoxia than in those from HBO-treated mice. HBO, hyperbaric oxygen; NOR, normoxia; HYP, hypoxia.

In order to investigate the effects of hyperbaric oxygen treatment on hypoxia and stemness, we performed a series of experiments. The results showed that Hypoxyprobe^™^-1, HIF1α, HIF2α, CD133, and CD15 were more highly expressed in U87 cells under hypoxia than under HBO treatment conditions (Figure 2A). HIF1α, HIF2α, CD133, and CD15 were highly expressed in the hypoxic microenvironment of glioma cells according to reverse transcription quantitative PCR (RT–qPCR) and western blotting results (Figure 2B; Supplementary Figure S1C). Moreover, the RT-qPCR and western blotting results also indicated that HIF1α, HIF2α, CD133, and CD15 were highly expressed in mouse tumor tissues under normoxia, while they were rarely or were less expressed in tumor tissues exposed to HBO (Figure 2C).

### Low expression of HIF1α/HIF2α promotes glioma cell proliferation and increases chemosensitivity

**Figure 3:**
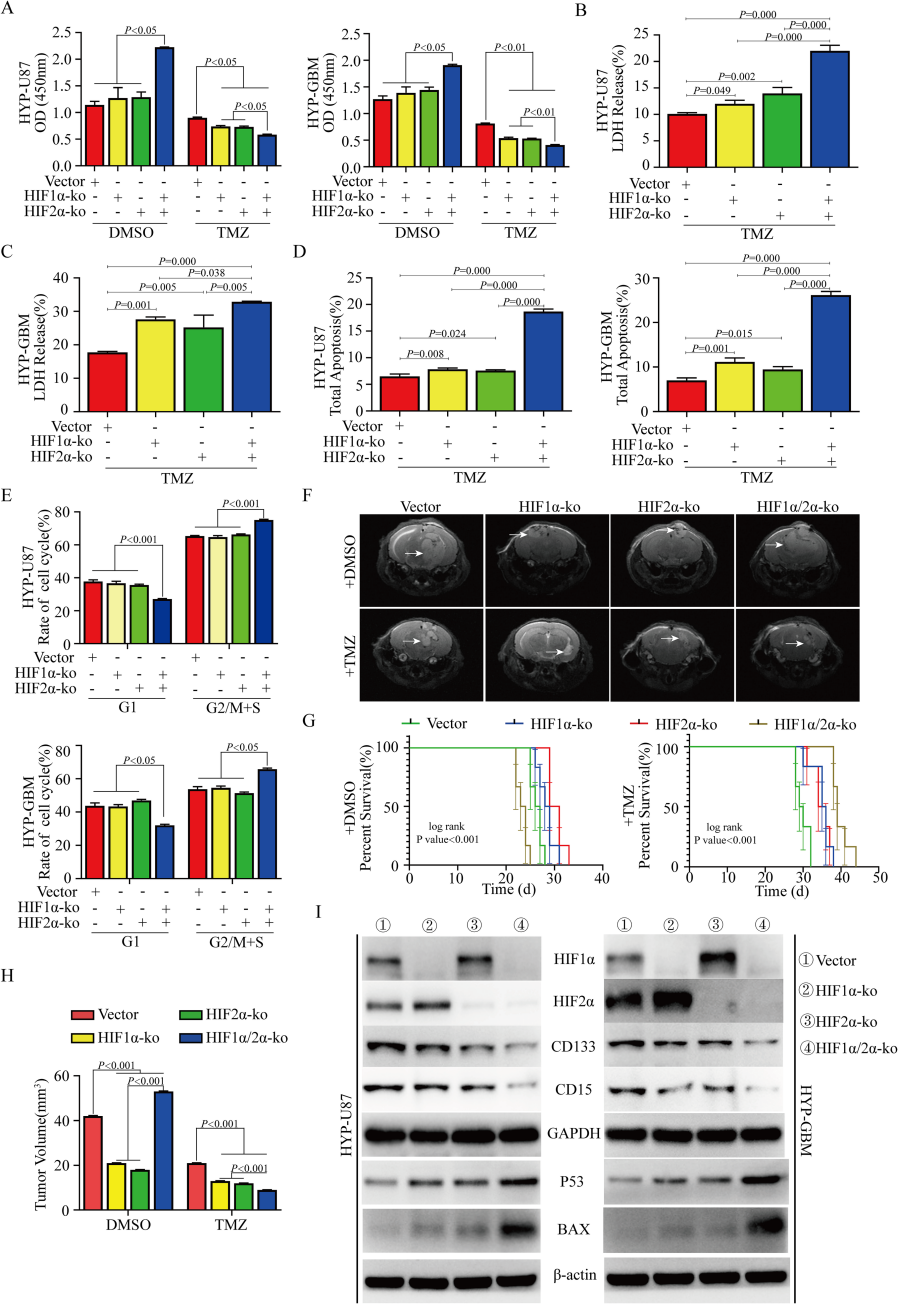
Knockout of HIF1α/HIF2α promotes glioma cell proliferation and chemosensitization. **(A)** CCK-8 assay showed that dual HIF1α and HIF2α knockout promoted the proliferation of U87 and GBM cells, but the proliferation of the HIF1α and/or HIF2α knockout groups was significantly inhibited after treatment with TMZ, and that of the dual-knockout group was significantly lower than that of the single-knockout group. **(B–D)** Knocking out HIF1α and/or HIF2α promoted LDH release and apoptosis in U87 and GBM cells under hypoxic conditions, and the LDH release and apoptosis were significantly promoted in the dual-knockout group compared with the single-knockout group. **(E)** HIF1α or HIF2α knockout under hypoxia had no significant effect on the cell cycle, but dual HIF1α and HIF2α knockout increased the number of U87 and GBM cells in the G2/M + S phase. **(F,H)** MRI revealed that HIF1α or HIF2α knockout reduced the tumor size compared with that in the empty vector group, but dual HIF1α and HIF2α knockout promoted the growth of mouse tumors. After treatment with TMZ, the tumor size significantly decreased compared with that in the empty vector group, and the tumor size in the dual-knockout group was lower than that in the single-knockout group. **(G)** Survival analysis revealed that dual HIF1α and HIF2α knockout combined with TMZ treatment significantly prolonged the survival time of mice. **(I)** The western blotting results showed that HIF1α and/or HIF2α knockout in U87 and GBM cells inhibited the expression of CD133 and CD15, and the expression in the dual HIF1α and HIF2α knockout group was lower than that in the single-knockout group, meanwhile, HIF1α and/or HIF2α knockout promoted the upregulation of apoptosis-related marker proteins, including p53 and BAX. HYP, hypoxia; TMZ, temozolomide; ko, knockout.

To further explore whether the decreased expression of HIF1α/HIF2α under HBO treatment conditions promoted glioma cell proliferation and chemosensitivity, CCK-8 assays and flow cytometry were used to detect the effect of HIF1α/HIF2α on glioma cell growth. The results showed that cell proliferation and cell cycle changes were not obvious (*p* > 0.05) after HIF1α or HIF2α knockout, but the cell proliferation of the dual HIF1α/HIF2α knockout group was significantly (*p* < 0.05) increased, and more cells were arrested in the G2/M + S phase (Figures 3A,E; Supplementary Figure S2A). After treatment with TMZ, the proliferation of the HIF1α-and/or HIF2α-knockout cells was significantly (*p* < 0.05) lower than that of the cells in the empty vector group, and the proliferation of the HIF1α/HIF2α dual-knockout cells was lower than that of the HIF1α or HIF2α single-knockout cells (Figure 3A; Supplementary Figure S2A). LDH concentration was measured in the culture medium, and the results revealed that TMZ treatment after HIF1α and/or HIF2α knockout significantly promoted LDH release, and LDH release in the dual-knockout group was significantly greater than that in the single-knockout group (Figures 3B,C; Supplementary Figure S2C). The flow cytometry results showed that the apoptosis rate of the HIF1α-and/or HIF2α-knockout groups was significantly (*p* < 0.05) greater than that of the empty vector group after TMZ treatment, and that of the dual-knockout group was significantly (*p* < 0.01) greater than that of the single-knockout groups (Figure 3D; Supplementary Figure S2B). MRI revealed that the tumor volume of the HIF1α-or HIF2α-knockout mice was significantly (*p* < 0.01) lower than that of the empty vector group, but the tumor volume of the dual HIF1α and HIF2α-knockout group was significantly (*p* < 0.01) greater than that of the empty vector group. After TMZ treatment, the tumor volumes of the HIF1α-and/or HIF2α-knockout groups were significantly (*p* < 0.01) reduced, and those of the dual-knockout group were lower than those of the single-knockout groups (Figures 3F,H). Further analysis revealed that the survival time of the HIF1α or HIF2α knockout groups was longer than that of the empty vector group, but the survival time of the dual-knockout group was significantly shorter than that of the empty vector group. After TMZ treatment, the survival time of the HIF1α-and/or HIF2α-knockout groups was longer than that of the empty vector group, and the survival time of the dual-knockout group was significantly (*p* < 0.01) longer than that of the single-knockout groups (Figure 3G). The GO analysis and KEGG pathway enrichment showed that HIF1α/2α associated with stemness, cell cycle, apoptosis, and chemosensitization (Supplementary Figure S3). Western blot analysis revealed that the protein expression of HIF2α was significantly increased, while the expression of the stem cell marker proteins CD133 and CD15 was significantly decreased after HIF1α knockout. HIF2α knockout significantly increased the protein expression of HIF1α and decreased the expression of the stem cell marker proteins CD133 and CD15. The expression of CD133 and CD15 was significantly lower in the dual HIF1α/HIF2α knockout group than in the HIF1α or HIF2α knockout group. After the knockout of HIF1α and/or HIF2α, the expression of apoptosis-related protein markers p53 and BAX were significantly upregulated. Moreover, when HIF1α and HIF2α were simultaneously knocked out, the expression levels of p53 and BAX were markedly higher than those in the individual knockout groups (Figure 3I; Supplementary Figure S2D).

### ABCG2 expression was decreased after hyperbaric oxygen treatment and was positively associated with HIF1α/HIF2α

**Figure 4:**
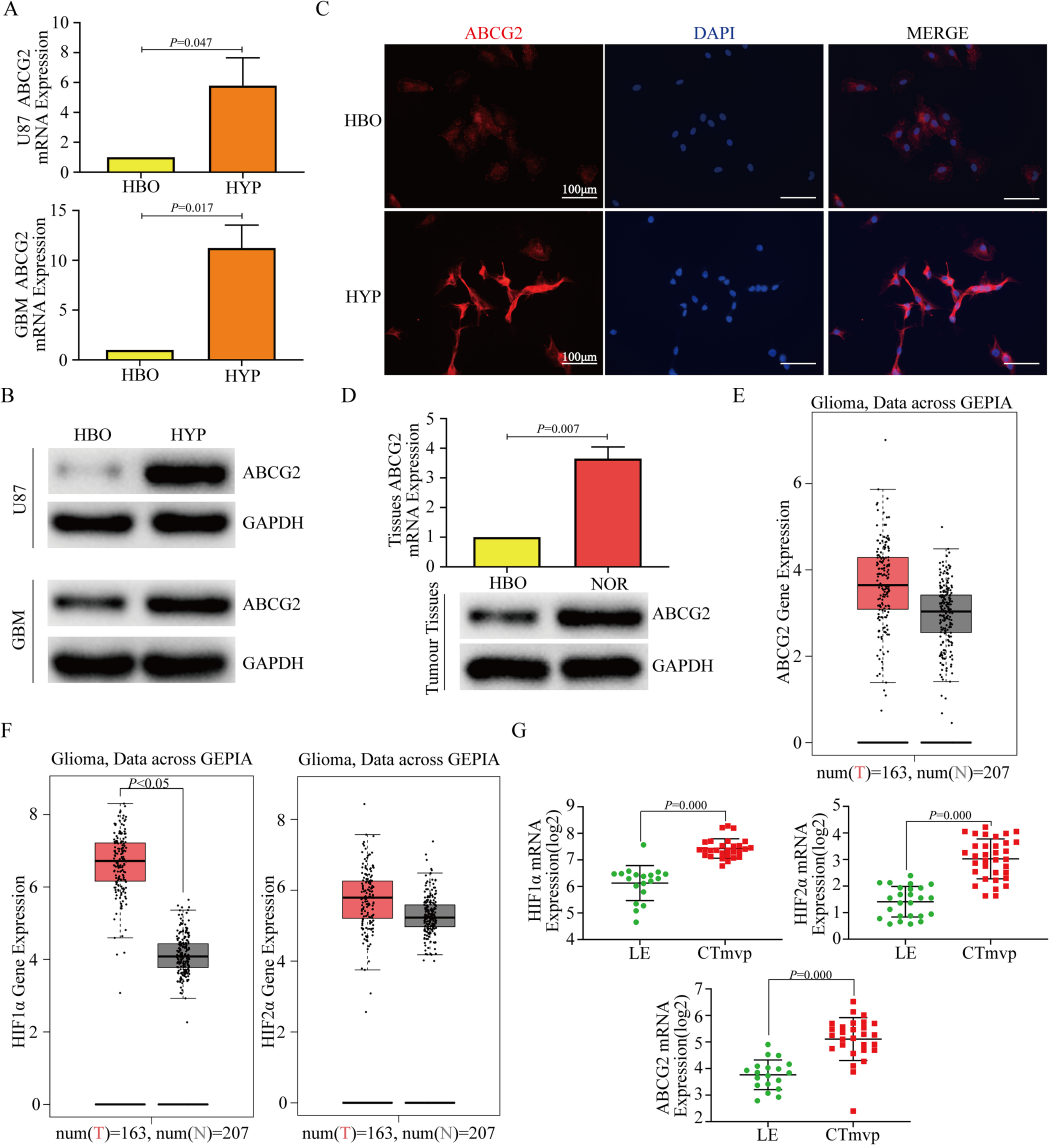
HBO inhibits the expression of ABCG2, and HIF1α and HIF2α may be involved. **(A,B)** Compared with hypoxia, HBO therapy inhibited the expression of ABCG2 in U87 and GBM cells. **(C)** The IF results showed that ABCG2 was more highly expressed in U87 cells under hypoxia than in HBO-treated cells. **(D)** The RT-qPCR and western blotting results showed that ABCG2 was more highly expressed in tumor tissues from mice reared under normoxia than in those from HBO-treated mice. **(E,F)** GEPIA database analysis showed that ABCG2 was highly expressed in GBM tissues, which was consistent with the expression trend of HIF1α/HIF2α. **(G)** ABCG2 expression was greater in CTmvp than in LE, similar to the expression patterns for HIF1α/HIF2α. HBO, hyperbaric oxygen; NOR, normoxia; HYP, hypoxia; CTmvp, microvascular proliferation region; LE, leading edge region.

To explore the expression differences of ABCG2 under hyperbaric oxygen and hypoxic conditions, we performed RT-qPCR, western blotting and Immunofluorescence. The results indicated that ABCG2 was highly expressed in the hypoxic microenvironment of glioma cells (Figures 4A–C; Supplementary Figure S1C). *In vivo* RT-qPCR and western blotting results indicated that ABCG2 was more highly expressed in mice reared under normoxia than in those reared under HBO treatment conditions (Figure 4D). To further investigate whether ABCG2 expression correlates with hypoxia-inducible factors (HIFs), we performed bioinformatics analyses. The GEPIA database showed that ABCG2 was highly expressed in GBM tissues, which was consistent with the trend of HIF1α/HIF2α expression (Figures 4E,F). Hypoxic area in glioma tissues always presented with more microvascular proliferation (Mvp) (Liu et al., 2020). According to the Ivy Glioblastoma Atlas project, ABCG2 expression was greater in the microvascular proliferation region (CTmvp) than in the leading edge region (LE), similar to the results for HIF1α/HIF2α (Figure 4G). The CGGA and GEPIA database results showed that both HIF1α and HIF2α are positively correlated with ABCG2 (Supplementary Figure S2E).

### HIF1α/HIF2α promotes the expression of ABCG2 through transcriptional regulation

**Figure 5:**
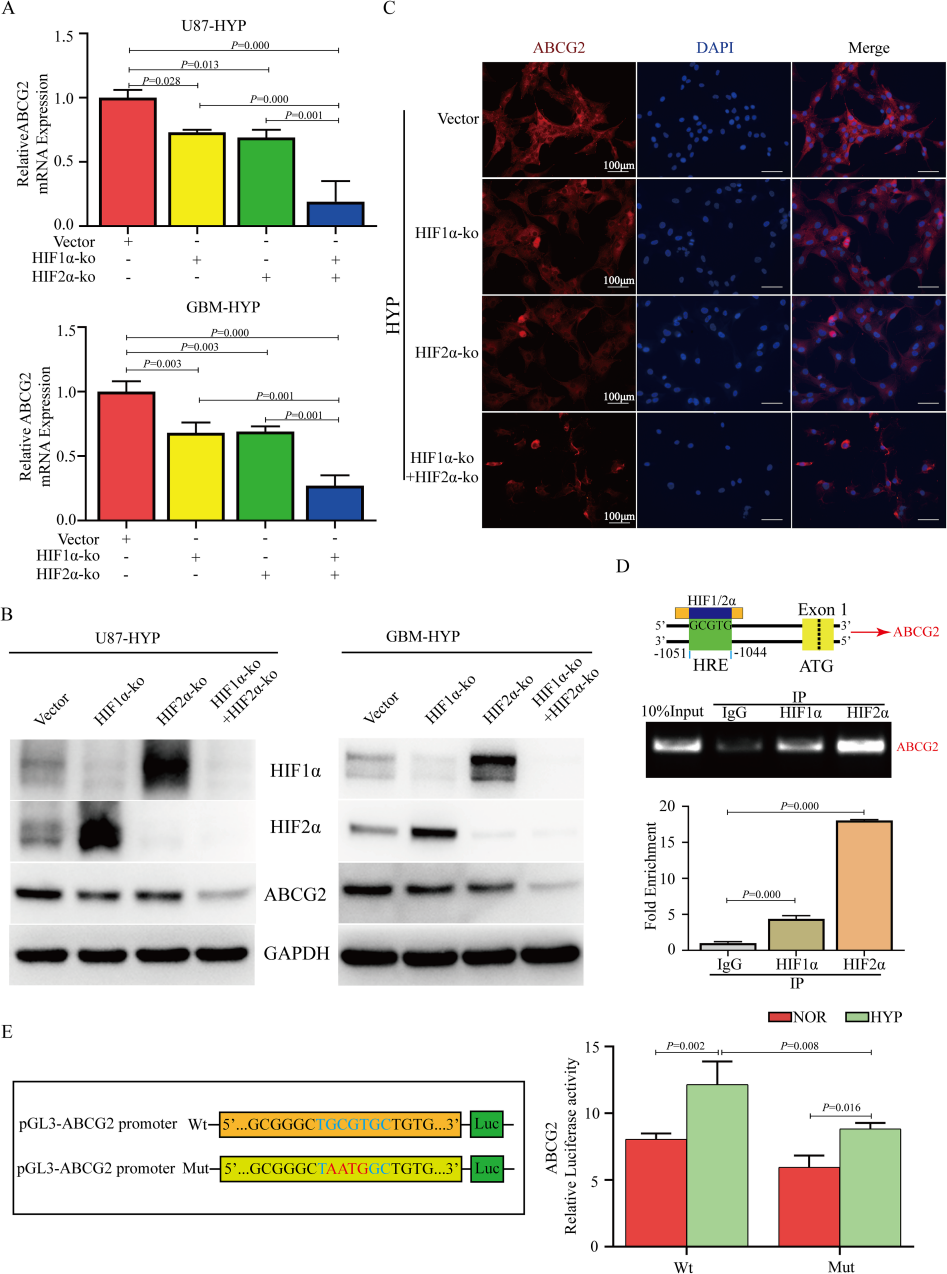
HIF1α and HIF2α transcriptionally regulate the expression of ABCG2. **(A,B)** Knockout of HIF1α or/and HIF2α inhibited the expression of ABCG2 in U87 and GBM cells under hypoxia, and its expression in the dual HIF1α and HIF2α knockout group was lower than that in the HIF1α or HIF2α knockout group. **(C)** IF results revealed that ABCG2 expression was inhibited by HIF1α and/or HIF2α knockout. **(D,E)** ChIP-qPCR and DLR revealed that HIF1/2α could target the HRE region of the ABCG2 promoter (−1,044 to −1,051) and promote transcription. HYP, hypoxia; ko, knockout; HRE, hypoxia response element; IP, immunoprecipitation; Wt, wild type; Mut, mutant.

To analyze the relationship between HIF1α/HIF2α and ABCG2, we conducted a series of experiments. The results demonstrated that the expression of ABCG2 in U87 and GBM cells was significantly decreased after dual HIF1α/HIF2α knockout and was lower than that in HIF1α or HIF2α knockout cells according to the RT-qPCR and western blotting results (Figures 5A,B). Knockout of HIF1α and/or HIF2α in U87 cells under hypoxia resulted in a decrease in ABCG2 expression, and the most significant decrease was observed after dual HIF1α/HIF2α knockout (Figure 5C). According to JASPAR^4^ database analysis, there is a core conserved sequence of the hypoxia response element (HRE) in the ABCG2 promoter region from −1,044 to −1,051. Based on these fingdings, primers were designed, and ChIP-qPCR was performed, the results revealed that HIF1/2α could target the HRE region of the ABCG2 promoter and promote the transcription of ABCG2 (Figure 5D). To elucidate the transcriptional regulation of ABCG2 by HIF1α and HIF2α, we performed a dual-luciferase reporter assay. The results revealed that hypoxia exposure induced a increase in transcriptional activity compared to normoxic controls (*p* < 0.05); crucially, mutations in the hypoxia-response elements (HREs) of the ABCG2 promoter (GCGT → AATG) significantly attenuated the activation under hypoxic conditions (*p* < 0.01) (Figure 5E).

### Hyperbaric oxygen increases glioma chemosensitivity by inhibiting ABCG2 expression

**Figure 6:**
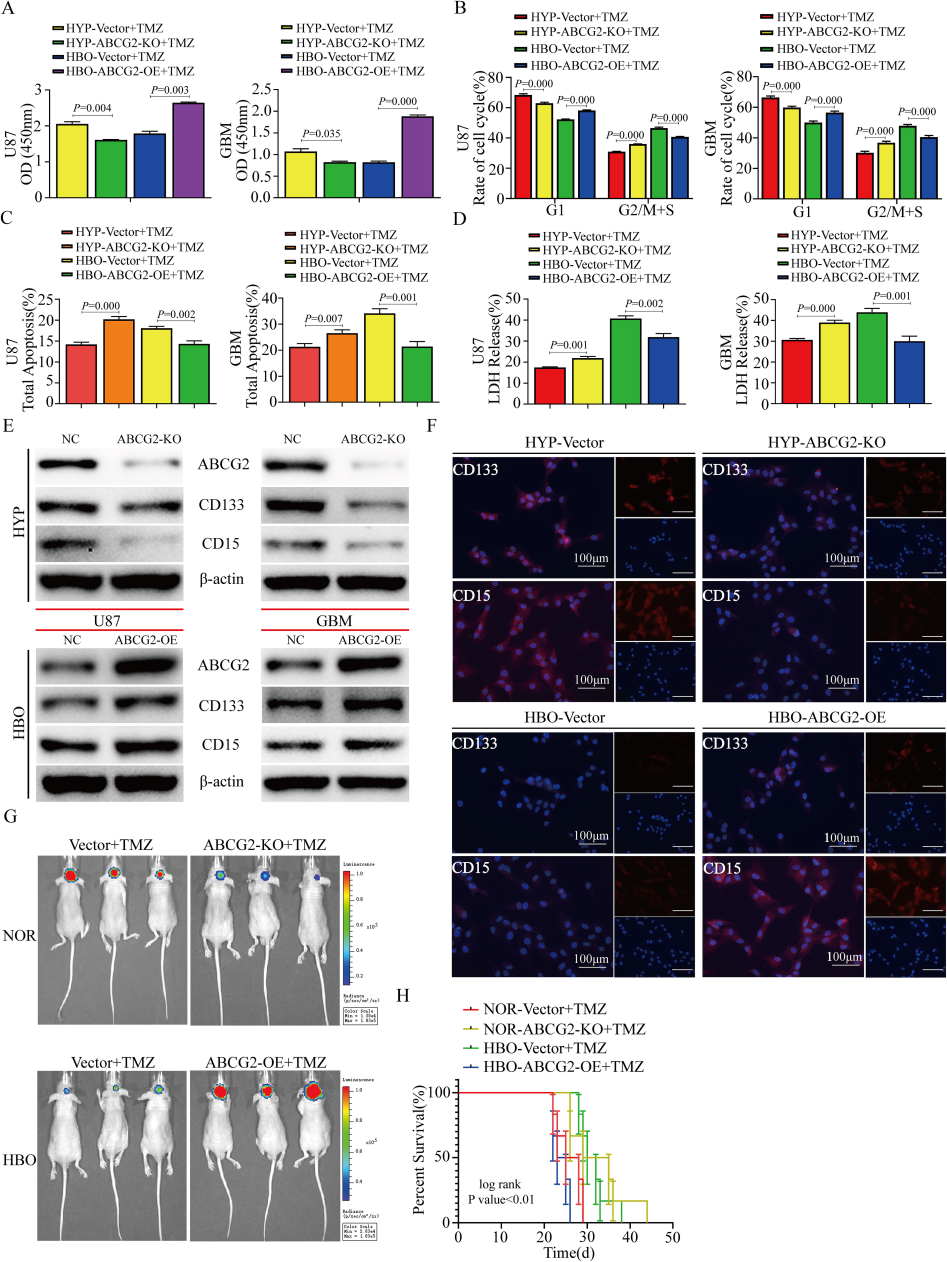
Hyperbaric oxygen promotes glioma chemosensitization by inhibiting ABCG2. **(A)** CCK-8 assay showed that the proliferation of U87 and GBM cells under hypoxia was decreased after ABCG2 knockout and TMZ treatment, and ABCG2 overexpression under HBO treatment promoted chemoresistance. **(B)** Under hypoxic conditions, knocking out ABCG2 and treatment with TMZ induced U87 and GBM cells arrest in G2/M + S phase, and a greater proportion of cells were in the G1 phase after ABCG2 overexpression and treatment with TMZ under HBO conditions. **(C,D)** The knockout of ABCG2 under hypoxia and TMZ treatment resulted in enhanced LDH release and increased cellular apoptosis, and the overexpression of ABCG2 under HBO conditions reduced LDH release and inhibited cell apoptosis even after treatment with TMZ. **(E,F)** Western blotting and IF revealed that the knockout of ABCG2 under hypoxic conditions resulted in the downregulation of stem cell marker proteins, whereas ABCG2 expression was increased upon the overexpression of ABCG2 under HBO conditions. **(G,H)** An *in vivo* study revealed that HBO combined with TMZ inhibited tumor growth and prolonged survival by inhibiting ABCG2 expression. HBO, hyperbaric oxygen; NOR, normoxia; HYP, hypoxia; TMZ, temozolomide; ko, knockout; OE, overexpression.

To further explore the function of ABCG2 under different oxygen concentrations in glioma cells, we performed gain-and loss-of-function experiments. The proliferation of U87, GBM and A172 cells decreased after ABCG2 knockout and treatment with TMZ under hypoxia, and more cells were also in the G2/M + S phase (Figures 6A,B; Supplementary Figure S4A). Proliferation significantly (*p* < 0.01) increased after ABCG2 overexpression and treatment with TMZ under HBO conditions; however, after the knockout of ABCG2 post-overexpression, cell proliferation decreased (Figures 6A,B; Supplementary Figure S4B). The rate of apoptosis significantly (*p* < 0.01) increased after ABCG2 knockout and treatment with TMZ under hypoxia, while the release rate of LDH increased significantly (*p* < 0.01) (Figures 6C,D; Supplementary Figure S4C). When ABCG2 was overexpressed and TMZ plus HBO treatment was administered, both apoptosis and the release rate of LDH decreased, however, after the knockout of ABCG2 post-overexpression, the apoptosis increased (Figures 6C,D; Supplementary Figure S4D). In order to investigate the relationship between ABCG2 and stemness, western blotting and immunofluorescence (IF) were performed. The results showed that the expression of the stem cell marker proteins CD133 and CD15 decreased after ABCG2 knockout under hypoxic conditions; however, following the overexpression of ABCG2 under hyperbaric oxygen (HBO) treatment, both CD133 and CD15 levels were elevated. On the basis of this observation, we performed additional rescue experiments, and the results indicated that the expression levels of CD133 and CD15 significantly decreased after the knockdown of ABCG2 post-overexpression, compared with the ABCG2 overexpression group (Figures 6E,F; Supplementary Figures S4E,F). We implanted U87 cells into mouse brains and found that the tumor fluorescence intensity of the ABCG2 knockout group was lower than that of the empty vector group when the mice were treated with TMZ (2 mg/kg) and reared under normoxic conditions, but the tumor fluorescence intensity of the ABCG2 overexpression group was greater than that of the empty vector group when the mice were treated with TMZ (2 mg/kg) and reared under HBO conditions (Figure 6G). Further survival analysis revealed that after treatment with TMZ (2 mg/kg), the survival time of the ABCG2 knockout group reared under normoxic conditions was the longest, and the overexpression of ABCG2 under HBO conditions significantly (*p* < 0.01) reduced the survival time (Figure 6H).

## Discussion

Glioblastoma, as the most serious malignant tumor in the brain, still has a poor prognosis despite significant progress in surgical treatment. It is generally believed that the hypoxic microenvironment is an important reason for the high malignancy and poor prognosis of gliomas due to its inhibition of tumor apoptosis and maintenance of tumor cell stemness. Hyperbaric oxygen (HBO) therapy is a promising intervention for ameliorating tumor hypoxic microenvironments. However, research on HBO treatment alone is limited, and the treatment effects are inconsistent. For instance, Arienti et al. (2021) reported that HBO inhibits tumor proliferation by improving the hypoxic microenvironment of G34 cells and inducing glucose metabolism reprogramming; while Wang et al. (2015) reported that HBO can promote the growth of GL261 cells. However, it is worth noting that they used different types of tumor cells. Our research confirmed that after HBO treatment, the tumors became larger, and the survival period was significantly shorter than that of the normoxic group. Cell experiments showed that HBO could significantly inhibit glioma cell apoptosis, and the cell cycle was blocked in the G2/M + S phase, thus promoting cell proliferation, which is consistent with the results of previous studies (Wang et al., 2015; Ding et al., 2015). Different tumor cells also exhibit varying responses to hyperbaric oxygen. Fortunately, there is a general consensus that HBO can strengthen the cell cycle blockade effect of temozolomide (TMZ) and increase the chemotherapy sensitivity of tumor cells, so HBO combined with TMZ treatment can significantly inhibit tumor growth (Yuen et al., 2023; Wang et al., 2022; Xie et al., 2018; Zeng et al., 2019). Our research showed that the volume of mouse tumors significantly decreased after treatment with the combination of HBO and TMZ, and the survival period was also longer than that in the normoxic combination with TMZ group. Compared to the hypoxia combined with TMZ group, the HBO therapy combined with TMZ group exhibited cell cycle arrest in the G2/M + S phase and promotion of glioma cell apoptosis, thereby inhibiting cell proliferation. A hypoxic microenvironment is an important reason for the malignant progression of gliomas and can inhibit apoptosis and maintain the stemness of glioma stem cells (GSCs) (Wang et al., 2021a; Wang et al., 2020; Heddleston et al., 2012). HIF1α/HIF2α serve as the primary effector molecules in hypoxic environments, and HIF1α/HIF2α protein accumulation and downstream target gene expression are influenced by changes in oxygen content (Semenza, 2001; Begagić et al., 2024). In mouse liver cancer models, hypoxia in tumor tissue leaded to the accumulation of HIF-1α, which suppressed the activation of the cGAS-STING signaling pathway, thereby affecting the induction of IFN-I and NFκB signaling by the chemotherapeutic drug teniposide; hyperbaric oxygen (HBO) therapy reduced the level of HIF-1α in tumor tissue, thereby alleviating the inhibitory effect of hypoxia on the cGAS-STING signaling pathway. The article also mentioned that knocking down the expression of HIF1α can similarly restore the activation of the cGAS-STING signaling pathway (Li K. et al., 2022). To date, most studies have focused on the expression of HIF1α under HBO conditions, with few examining HIF2α under the same conditions. To further elucidate the expression of HIF1α/HIF2α under HBO conditions, we conducted both *in vitro* and *in vivo* experiments. These results confirmed that HBO significantly decreased HIF1α/HIF2α expression, which is consistent with previous research findings (Zembrzuska et al., 2019). Simultaneously, it was found that the expression of CD133 and CD15, which are markers of glioma stem cells (GSCs), significantly decreased after hyperbaric oxygen treatment. Some articles have indicated that hyperbaric oxygen can reduce the stemness of cancer cells, thereby increasing their sensitivity to chemotherapy (Yuen et al., 2023; Song et al., 2020). Hence, we wondered whether the HBO-induced reduction in HIF1α/HIF2α expression could be a key factor in the inhibition of stemness in glioblastoma. Experimental results showed insignificant cell growth differences after the knockout of either HIF1α or HIF2α alone; however, simultaneous knockout of HIF1α and HIF2α resulted in cell cycle arrest at the G2/M + S phase, a significant increase in cell proliferation, and a notable reduction in mouse survival time. Interestingly, *in vivo* experiments revealed significantly smaller tumor volumes and longer survival times after the individual knockout of either HIF1α or HIF2α compared to those in the control group, which may be due to microenvironmental differences between *in vivo* and *in vitro* studies. After knocking out HIF1α and/or HIF2α and administering TMZ, there was an increase in cell apoptosis and a significant decrease in cell growth, especially in the dual-knockout group. Previous studies have proven that hypoxic microenvironments can maintain the stemness of GSCs via HIF1α and HIF2α (Wang et al., 2021a; Wang et al., 2020; Wang et al., 2022; Li Y. P. et al., 2022; Ling et al., 2020; Wang et al., 2021b; Ahmed et al., 2018). Further western blot analysis confirmed that the protein expression of CD133 and CD15 decreased significantly after the knockout of HIF1α and/or HIF2α, and the protein expression in the dual-knockout group was significantly lower than that in the single-knockout groups. According to these results, we concluded that HBO promotes the proliferation and chemosensitization by decreasing HIF1α and HIF2α expression to inhibit stemness.

**Figure 7:**
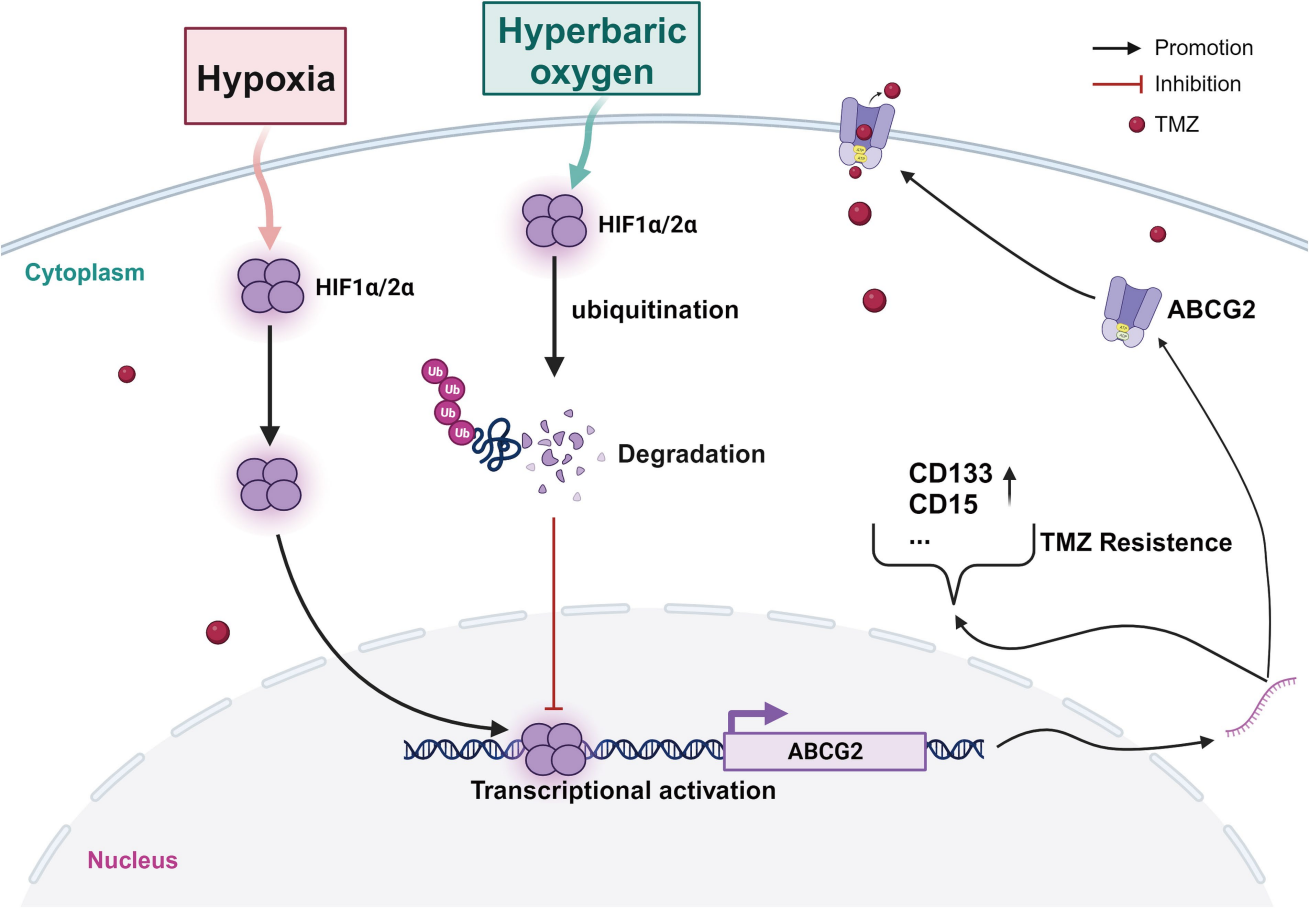
Schematic of the mechanism. Under HBO treatment, the accumulation of HIF1α and HIF2α proteins is reduced through ubiquitination, which can transcriptionally regulate the expression of ABCG2, thereby affecting the maintenance of stem cell stemness, promoting proliferation, and increasing chemotherapy sensitivity.

ABCG2 expression is associated with glioma cell stem-like properties, including self-renewal and differentiation potential, which are reasons for tumor recurrence and resistance (Singh et al., 2004; Wee et al., 2016). To date, there have been no reports on the effect of HBO on ABCG2 expression. *In vitro* and *in vivo* experiments revealed that HBO significantly inhibited the transcription and translation of ABCG2. Hence, whether the decrease in ABCG2 expression under HBO conditions is related to HIF1/2α requires further verification. The GEPIA database revealed that both HIF1/2α and ABCG2 are highly expressed in tumors. Hypoxic areas in gliomas always present with increased microvascular proliferation (Mvp) (Liu et al., 2020). According to the Ivy Glioblastoma Atlas project, we found that HIF1α, HIF2α, and ABCG2 expression was greater in the microvascular proliferation region (CTmvp) than in the leading-edge region (LE). Correlation analysis revealed a positive correlation between HIF1/2α and ABCG2 expression. Knocking out of HIF1α and/or HIF2α inhibited ABCG2 expression, and the expression with dual knockout was significantly lower than that with single knockouts. He et al. (2016) revealed that HIF1α binds to the ABCG2 promoter in Canpan-2 cells to regulate its transcription. However, the relationship between HIF2α and ABCG2 has received limited attention in the literature. The ChIP-qPCR and DLR results confirmed that HIF1/2α could target the −1,051 to −1,044 region of the ABCG2 promoter, thereby regulating its expression. To explore the role of ABCG2 in glioma progression, we conducted further experiments. After ABCG2 knockout under hypoxic conditions, TMZ treatment led to cell cycle arrest at the G2/M + S phase, increased apoptosis, decreased cell proliferation, and decreased expression of the stem cell marker proteins CD133 and CD15. However, after the overexpression of ABCG2 under HBO conditions, TMZ treatment led to cell cycle arrest in the G1 phase, decreased apoptosis, promoted cell proliferation, and increased the expression of the stem cell marker proteins CD133 and CD15. As reported by Xu et al., overexpression of ABCG2 reversed the inhibitory effect of SMAR1 overexpression on the stemness of OS cells, and the SMAR1/ABCG2 axis was found to positively regulate the chemosensitivity of OS cells (Xu et al., 2021). Moreover, ABCG2, a drug efflux transporter, plays an important role in tumor multidrug resistance (Dean et al., 2022; Dean et al., 2005; Gong et al., 2011). Our *in vivo* experiments also confirmed this finding. In conclusion, ABCG2 can promote the stemness of tumor cells and combat multidrug resistance to achieve chemotherapy resistance in glioma. In brief, the proposed mechanism is as follows (Figure 7). Under hypoxic conditions, HIF1/2α is highly expressed, but under HBO conditions, HIF1/2α expression is significantly suppressed, the cell cycle is arrested in the G2/M + S phase, apoptosis is suppressed, and proliferation is promoted. HIF1/2α, as a transcription factor of ABCG2, can target the ABCG2 promoter and promote its expression. ABCG2 serves as a drug efflux transporter and plays a significant role in tumor multidrug resistance and stemness maintenance. HBO reduced ABCG2 expression, thereby inhibiting tumor cell stemness and drug efflux to increase chemotherapy sensitivity. In conclusion, HBO can affect the progression of glioma by regulating ABCG2 expression through HIF1/2α.

The mechanisms by which hyperbaric oxygen (HBO) influences tumor progression are notably intricate. In addition to the proposed regulation of tumor stem cells, which affects glioma progression, there exist hypoxia-independent pathways, such as reactive oxygen species (ROS) signaling (Schottlender et al., 2021; Li et al., 2023) and metabolic reprogramming (Arienti et al., 2021; Ma et al., 2022), that significantly contribute to the action mechanisms of HBO. Numerous recent and historical studies have demonstrated that HBO can inhibit and reduce cancer growth in specific types of malignancies, including breast cancer (Xiong et al., 2023). Conversely, HBO has also been shown to promote the proliferation of certain tumor cell lines, such as GL261 (Wang et al., 2015). These findings underscore that tumors exhibit variable responses to HBO treatment. Consequently, randomized trials assessing HBO alone or in combination with other therapies (including chemotherapy and radiotherapy) across different cancer types and even subtypes are warranted. Furthermore, it is essential to acknowledge the disadvantages and limitations associated with this treatment modality. First, hyperbaric oxygen therapy may induce adverse reactions, including oxygen seizures and barotrauma (Heyboer et al., 2017). Second, owing to the non-specific nature of oxygen delivery, HBO therapy may fail to effectively target hypoxic tumor regions, potentially resulting in oxygen toxicity as a notable side effect.

## Data Availability

The datasets presented in this study can be found at: https://www.ncbi.nlm.nih.gov/, GSE295278.

## Footnotes

^1^ http://gepia.cancer-pku.cn/detail.php

^2^ http://cgga.org.cn/index.jsp

^3^ https://glioblastoma.alleninstitute.org/

^4^ http://jaspar.genereg.net/

## Supplementary material

SUPPLEMENTARY FIGURE S1 Hyperbaric oxygen (HBO) promotes glioma cell proliferation but promotes chemosensitization in A172 cells. **(A)** CCK-8 assay revealed that HBO promoted A172 cell proliferation, but proliferation was significantly reduced when HBO was combined with TMZ. **(B)** HBO inhibited A172 cell apoptosis compared with that under hypoxia conditions, but the total percentage of apoptosis cells significantly increased after treatment with both HBO and TMZ. **(C)** Western blot analysis revealed that, compared with hyperbaric oxygen conditions, hypoxia promoted the protein expression of HIF1α, HIF2α, CD133, CD15, and ABCG2. HBO, hyperbaric oxygen; HYP, hypoxia; TMZ, temozolomide.

SUPPLEMENTARY FIGURE S2 Knockout of HIF1α/HIF2α promotes glioma cell proliferation and chemosensitization in A172 cells. **(A)** Under HYP treatment, CCK-8 assay revealed that dual HIF1α and HIF2α knockout promoted the proliferation of A172 cells, but the proliferation of the HIF1α- and/or HIF2α-knockout groups was significantly inhibited after treatment with TMZ, and that of the dual-knockout group was significantly lower than that of the single-knockout group. **(B)** The flow cytometry results showed that the apoptosis rate of the HIF1α- and/or HIF2α-knockout groups was significantly greater than that of the empty vector group after TMZ treatment, and that of the dual-knockout group was significantly greater than that of the single-knockout groups. **(C)** LDH assays revealed that TMZ treatment after HIF1α and/or HIF2α knockout significantly promoted LDH release, and LDH release in the dual-knockout group was significantly greater than that in the single-knockout group. **(D)** Western blot analysis revealed that the protein expression of the stem cell marker protein CD133, CD15, and ABCG2 were significantly decreased after HIF1α and/or HIF2α knockout. **(E)** According to the CGGA and GEPIA database, both HIF1α and HIF2α are positively correlated with ABCG2. HYP, hypoxia; TMZ, temozolomide; ko, knockout.

SUPPLEMENTARY FIGURE S3 mRNA-Seq showed that HIF1α/2α associated with stemness, cell cycle, apoptosis, and chemosensitization. **(A)** GO analysis presented that stemness, cell cycle, apoptosis, and proliferation had significant difference in HIFs knockout groups compared with empty vector group. **(B)** Compared with empty vector group, the cell cycle, apoptosis, and proliferation had significant difference in HIFs knockout groups from KEGG pathway enrichment analysis.

SUPPLEMENTARY FIGURE S4 The impact of ABCG2 on glioma progression. **(A)** Under hypoxic conditions, after the knockout of ABCG2 in A172 cells followed by treatment with TMZ, CCK-8 assays were conducted to assess cell proliferation, and the results demonstrated that cell proliferation was significantly inhibited. **(B)** Under hyperbaric oxygen conditions, following the overexpression of ABCG2 and treatment with TMZ, CCK-8 assays revealed an increase in cell proliferation. Subsequent knockout of ABCG2 after its overexpression resulted in a significant decrease in cell proliferation compared to the overexpression group. **(C)** The flow cytometry results showed that the apoptosis rate of the ABCG2-knockout group was significantly greater than that of the empty vector group after TMZ treatment. **(D)** The apoptosis rate of ABCG2-overexpression group was significantly lower than that of the empty vector group after TMZ treatment, however, subsequent knockout of ABCG2 after its overexpression resulted in a significant increase compared with that of the overexpression group. **(E)** Western blot analysis revealed that the protein expression of the stem cell marker protein CD133 and CD15 were significantly decreased after ABCG2 knockout. **(F)** Western blot analysis revealed that the protein expression of CD133 and CD15 were significantly increased after ABCG2 overexpression, however, knockout of ABCG2 after its overexpression resulted in a significant decrease compared with that in the overexpression group. HBO, hyperbaric oxygen; HYP, hypoxia; TMZ, temozolomide; ko, knockout; OE, overexpression; ns, no significance; Con, control.
