# Supplementary figures and images for: Hyperbaric oxygen promotes both the proliferation and chemosensitization of glioblastoma cells by inhibiting HIF1α/HIF2α-ABCG2
Source: Front Mol Neurosci. 2025 Apr 30;18:1584407. doi: 10.3389/fnmol.2025.1584407 (PMC12075184; doi:10.3389/fnmol.2025.1584407)

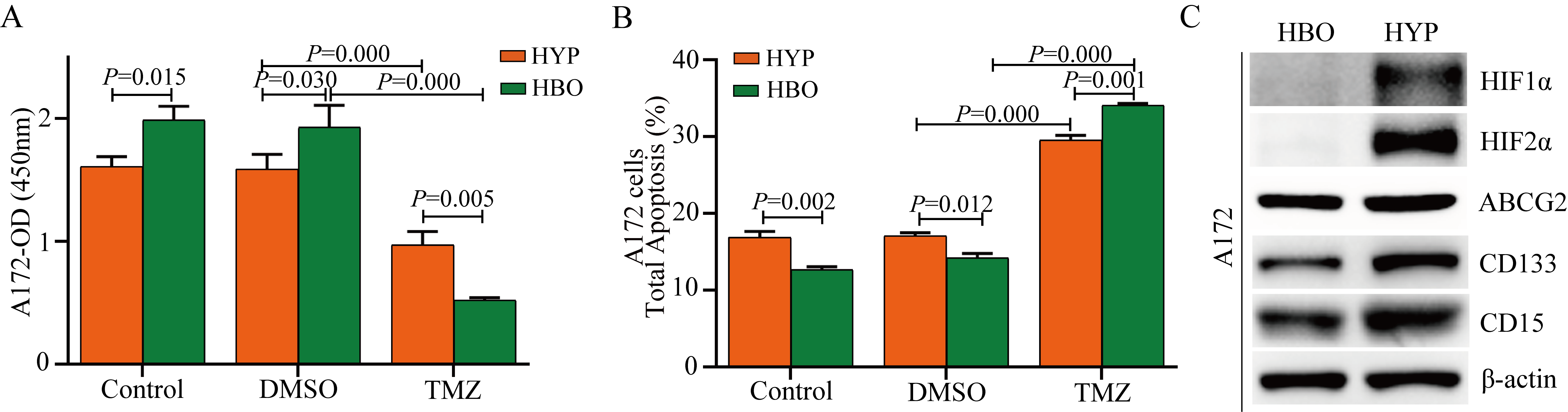

Supplement: SUPPLEMENTARY FIGURE S1 — Hyperbaric oxygen (HBO) promotes glioma cell proliferation but promotes chemosensitization in A172 cells. (A) CCK-8 assay revealed that HBO promoted A172 cell proliferation, but proliferation was significantly reduced when HBO was combined with TMZ. (B) HBO inhibited A172 cell apoptosis compared with that under hypoxia conditions, but the total percentage of apoptosis cells significantly increased after treatment with both HBO and TMZ. (C) Western blot analysis revealed that, compared with hyperbaric oxygen conditions, hypoxia promoted the protein expression of HIF1α, HIF2α, CD133, CD15, and ABCG2. HBO, hyperbaric oxygen; HYP, hypoxia; TMZ, temozolomide. [file Image_1.TIF]

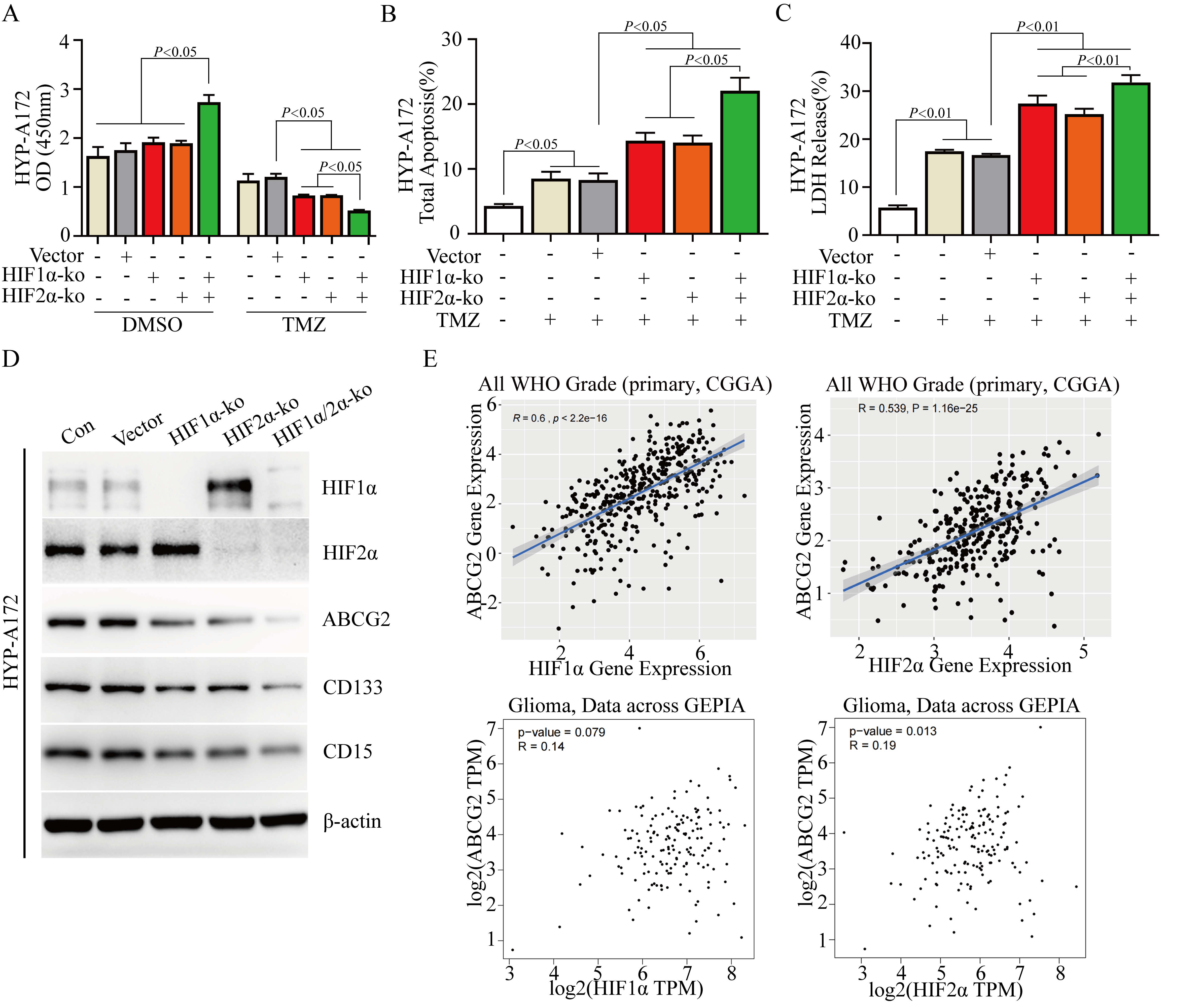

Supplement: SUPPLEMENTARY FIGURE S2 — Knockout of HIF1α/HIF2α promotes glioma cell proliferation and chemosensitization in A172 cells. (A) Under HYP treatment, CCK-8 assay revealed that dual HIF1α and HIF2α knockout promoted the proliferation of A172 cells, but the proliferation of the HIF1α- and/or HIF2α-knockout groups was significantly inhibited after treatment with TMZ, and that of the dual-knockout group was significantly lower than that of the single-knockout group. (B) The flow cytometry results showed that the apoptosis rate of the HIF1α- and/or HIF2α-knockout groups was significantly greater than that of the empty vector group after TMZ treatment, and that of the dual-knockout group was significantly greater than that of the single-knockout groups. (C) LDH assays revealed that TMZ treatment after HIF1α and/or HIF2α knockout significantly promoted LDH release, and LDH release in the dual-knockout group was significantly greater than that in the single-knockout group. (D) Western blot analysis revealed that the protein expression of the stem cell marker protein CD133, CD15, and ABCG2 were significantly decreased after HIF1α and/or HIF2α knockout. (E) According to the CGGA and GEPIA database, both HIF1α and HIF2α are positively correlated with ABCG2. HYP, hypoxia; TMZ, temozolomide; ko, knockout. [file Image_2.TIF]

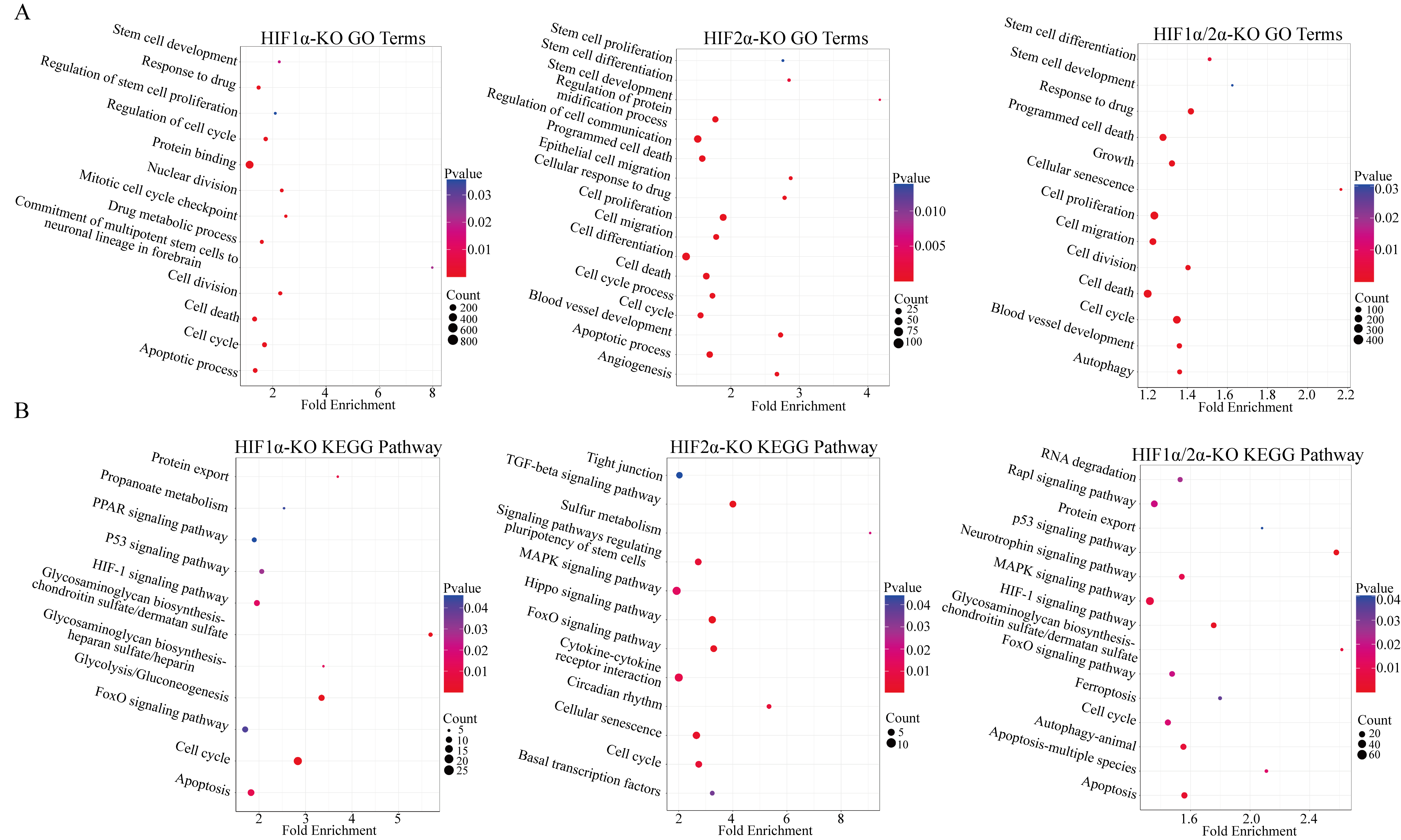

Supplement: SUPPLEMENTARY FIGURE S3 — mRNA-Seq showed that HIF1α/2α associated with stemness, cell cycle, apoptosis, and chemosensitization. (A) GO analysis presented that stemness, cell cycle, apoptosis, and proliferation had significant difference in HIFs knockout groups compared with empty vector group. (B) Compared with empty vector group, the cell cycle, apoptosis, and proliferation had significant difference in HIFs knockout groups from KEGG pathway enrichment analysis. [file Image_3.TIF]

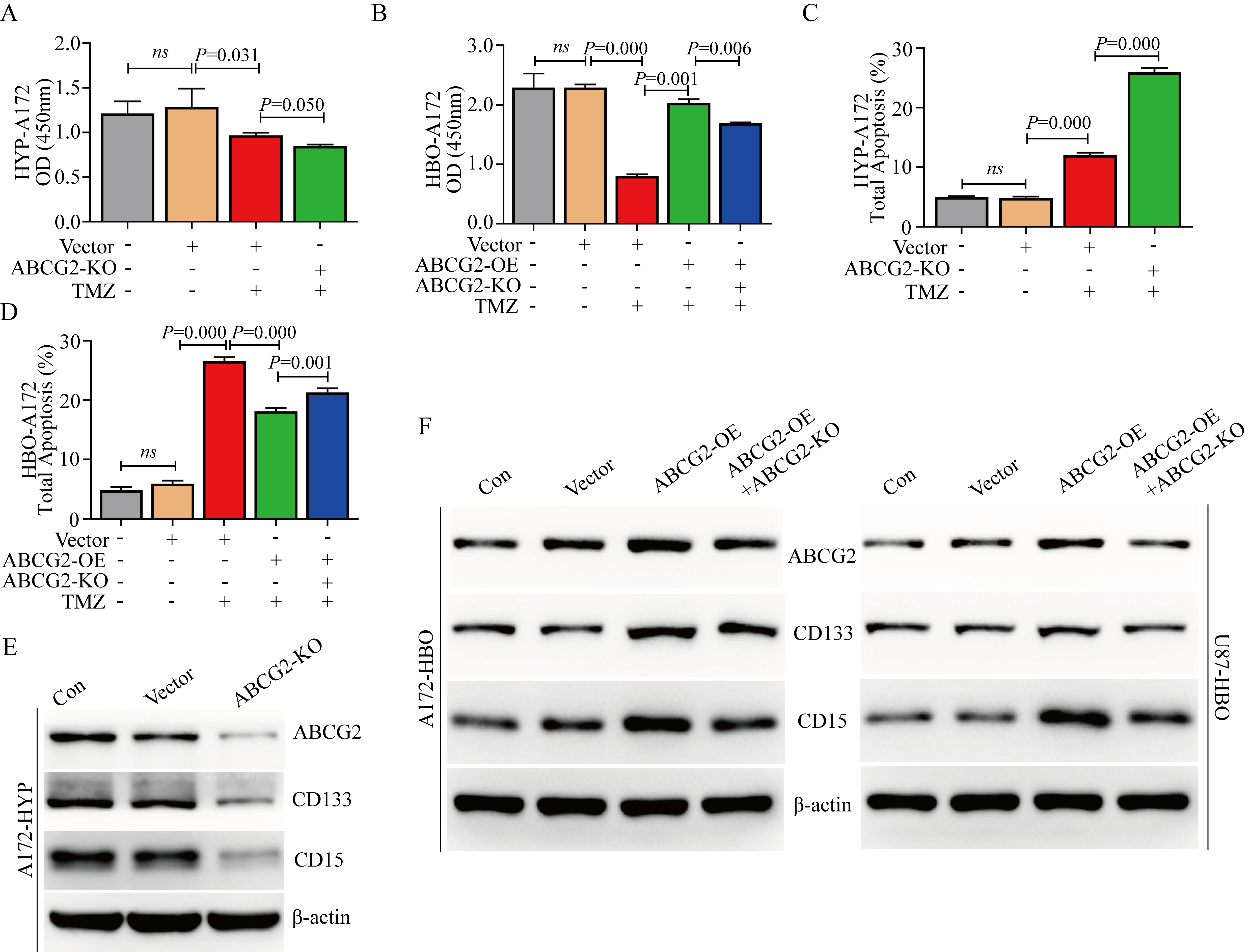

Supplement: SUPPLEMENTARY FIGURE S4 — The impact of ABCG2 on glioma progression. (A) Under hypoxic conditions, after the knockout of ABCG2 in A172 cells followed by treatment with TMZ, CCK-8 assays were conducted to assess cell proliferation, and the results demonstrated that cell proliferation was significantly inhibited. (B) Under hyperbaric oxygen conditions, following the overexpression of ABCG2 and treatment with TMZ, CCK-8 assays revealed an increase in cell proliferation. Subsequent knockout of ABCG2 after its overexpression resulted in a significant decrease in cell proliferation compared to the overexpression group. (C) The flow cytometry results showed that the apoptosis rate of the ABCG2-knockout group was significantly greater than that of the empty vector group after TMZ treatment. (D) The apoptosis rate of ABCG2-overexpression group was significantly lower than that of the empty vector group after TMZ treatment, however, subsequent knockout of ABCG2 after its overexpression resulted in a significant increase compared with that of the overexpression group. (E) Western blot analysis revealed that the protein expression of the stem cell marker protein CD133 and CD15 were significantly decreased after ABCG2 knockout. (F) Western blot analysis revealed that the protein expression of CD133 and CD15 were significantly increased after ABCG2 overexpression, however, knockout of ABCG2 after its overexpression resulted in a significant decrease compared with that in the overexpression group. HBO, hyperbaric oxygen; HYP, hypoxia; TMZ, temozolomide; ko, knockout; OE, overexpression; ns, no significance; Con, control. [file Image_4.TIF]
