# Supplementary material for: Hyperbaric oxygen promotes both the proliferation and chemosensitization of glioblastoma cells by inhibiting HIF1α/HIF2α-ABCG2
Source: Front Mol Neurosci. 2025 Apr 30;18:1584407. doi: 10.3389/fnmol.2025.1584407 (PMC12075184; doi:10.3389/fnmol.2025.1584407)
Supplement: Supplementary file 5 [file Table_1.DOCX]

Table S1 Primary antibodies used for immunofluorescence analysis

| Antigens | Manufacturer | Catalogue numbers | Application |
| --- | --- | --- | --- |
| HIF1α | abcam | ab179483 | 1:500 |
| HIF2α | NOVUS | NB100-132SS | 1:200 |
| ABCG2 | Proteintech | 27286-1-AP | 1:300 |
| CD133 | Proteintech | 18470-1-AP | 1:300 |
| CD15 | NOVUS | NB100-1831 | 1:200 |
