# Supplementary material for: Hyperbaric oxygen promotes both the proliferation and chemosensitization of glioblastoma cells by inhibiting HIF1α/HIF2α-ABCG2
Source: Front Mol Neurosci. 2025 Apr 30;18:1584407. doi: 10.3389/fnmol.2025.1584407 (PMC12075184; doi:10.3389/fnmol.2025.1584407)
Supplement: Supplementary file 6 [file Table_2.DOCX]

Table S2 Primary antibodies used for western blotting

| Antigens | Manufacturer | Catalogue numbers | Application |
| --- | --- | --- | --- |
| HIF1α | abcam | ab179483 | 1:1000 |
| HIF2α | abcam | ab207607 | 1:1000 |
| ABCG2 | Proteintech | 27286-1-AP | 1:1000 |
| CD133 | Proteintech | 18470-1-AP | 1:2000 |
| CD15 | NOVUS | NB100-1831 | 2ug/ml |
| GAPDH | Sangon Biotech | D110016-0200 | 1:5000 |
| β-actin | Sangon Biotech | [D191047-0100](https://store.sangon.com/productDetail?productInfo.code=D191047-0100) | 1:5000 |
| P53 | Proteintech | 60283-2-Ig | 1:5000 |
| BAX | Proteintech | 60267-1-Ig | 1:5000 |
