# Supplementary material for: Hyperbaric oxygen promotes both the proliferation and chemosensitization of glioblastoma cells by inhibiting HIF1α/HIF2α-ABCG2
Source: Front Mol Neurosci. 2025 Apr 30;18:1584407. doi: 10.3389/fnmol.2025.1584407 (PMC12075184; doi:10.3389/fnmol.2025.1584407)
Supplement: Supplementary file 7 [file Table_3.DOCX]

Table S3 Sequences of primers used for RT-qPCR

| HIF1α | Forward(5'-3') | TGTACCCTAACTAGCCGAGGA |
| --- | --- | --- |
|  | Reverse(5'-3') | AGCACCAAGCAGGTCATAGG |
| HIF2α | Forward(5'-3') | GCCCCTGCTGTCCTGCCTCATCATC |
|  | Reverse(5'-3') | CGGCCAAGCAGCTCCTCAGGG |
| ABCG2 | Forward(5'-3') | CCCGCGACAGCTTCCAATGAC |
|  | Reverse(5'-3') | CGAAGATTTGCCTCCACCTGTG |
| β-Actin | Forward(5'-3') | ACCCGCCGCCAGCTCACC |
|  | Reverse(5'-3') | GGGGGGCACGAAGGCTCATC |
