# Supplementary material for: Hyperbaric oxygen promotes both the proliferation and chemosensitization of glioblastoma cells by inhibiting HIF1α/HIF2α-ABCG2
Source: Front Mol Neurosci. 2025 Apr 30;18:1584407. doi: 10.3389/fnmol.2025.1584407 (PMC12075184; doi:10.3389/fnmol.2025.1584407)
Supplement: Supplementary file 8 [file Table_4.DOCX]

Table S4 Sequences of primers used for ChIP-qPCR

| ABCG2 | Forward(5'-3') | CCAAAGGACTATAAATCATGCTGCT |
| --- | --- | --- |
|  | Reverse(5'-3') | TTTCCAATTTCATCCATGTCCCTAC |
| GAPDH | Forward(5'-3') | GATTCCACCCATGGCAAATTC |
|  | Reverse(5'-3') | CTGGAAGATGGTGATGGGATT |
